# Primary left intrathoracic extrapulmonary trans-diaphragmatic hydatid cyst causing eventration: first case in literature

**DOI:** 10.1093/jscr/rjae458

**Published:** 2024-07-16

**Authors:** Saddik Haddad, George Bashour, Hussein Kaada, Samer Rajab, Moatasem Hussein Al-Janabi, Zuheir Alshehabi

**Affiliations:** Faculty of Medicine, Tishreen University, GRF4+3WH, Latakia 2230, Syria; Cancer Research Center, Tishreen University Hospital, GRF3+R8F, Latakia 2230, Syria; Faculty of Medicine, Tishreen University, GRF4+3WH, Latakia 2230, Syria; Cancer Research Center, Tishreen University Hospital, GRF3+R8F, Latakia 2230, Syria; Department of Thoracic Surgery, Tishreen University Hospital, GRF3+R8F, Latakia 2230, Syria; Department of Thoracic Surgery, Tishreen University Hospital, GRF3+R8F, Latakia 2230, Syria; Department of Pathology, Tishreen University Hospital, GRF3+R8F, Latakia 2230, Syria; Cancer Research Center, Tishreen University Hospital, GRF3+R8F, Latakia 2230, Syria; Department of Pathology, Tishreen University Hospital, GRF3+R8F, Latakia 2230, Syria

**Keywords:** hydatid cyst, intrathoracic extrapulmonary hydatid cysts, case report

## Abstract

Hydatidosis is a zoonotic parasitic disease caused by the cystic stage of Echinococcus species. Intrathoracic extrapulmonary hydatid cysts causing eventration are very rare. Here, we report a case of a 62-year-old female who presented with chest pain, intermittent coughing, general weakness, and fever. On auscultation, there were diminished respiratory sounds at the base of the left lung. A computed tomography scan showed a cystic formation with an ambiguous location involving the left lower thorax and the left hypochondrium. Complete surgical resection is the standard treatment for intrathoracic extrapulmonary hydatid cysts. Due to the direct bordering of the cyst with the pericardium in the left cadiophrenic angle, a cystotomy and evacuation of the cystic cavity were performed, followed by washing it with povidone and hyperosmolar saline. The location of the hydatid cyst has an important role in determining the surgical approach, as the unusual location could affect the possibility of radically removing the cyst.

## Introduction

Hydatidosis is a zoonotic parasitic disease caused by the cystic stage of *Echinococcus* species, which is commonly known as hydatid cyst (HC) [1]. Its prevalence is very high in the Mediterranean region [1, 2]. *Echinococcus granulosus* species is responsible for >95% of cases [2]. The most commonly affected organ is the liver, followed by the lungs [3]. Intrathoracic extrapulmonary HCs are very rare, with an occurrence rate of 7.4% of total thoracic HCs [4].

Here, we report a one-of-a-kind case of a primary left intrathoracic extrapulmonary trans-diaphragmatic HC causing eventration. To the best of our knowledge, this is the first case reported in the literature.

## Case report

A 62-year-old female patient presented to our thoracic surgery department due to repeated attacks of chest pain, hiccups, intermittent coughing, general weakness, and bouts of fever. On physical examination, the chest wall was symmetrical on both sides; there was no tenderness on palpation, and on auscultation, there were diminished respiratory sounds at the base of the left lung. No significant history was reported.

The abdominal echo showed an 18-cm cystic formation in the left hypochondrium, extending to the thoracic cavity. The mass is pressing on the left hepatic lobe and contains several cystic formations. The cyst has direct contact with the pericardium.

Labs were normal, and the *Echinococcus granulosus* antibody test was negative (1/80).

**Figure 1:**
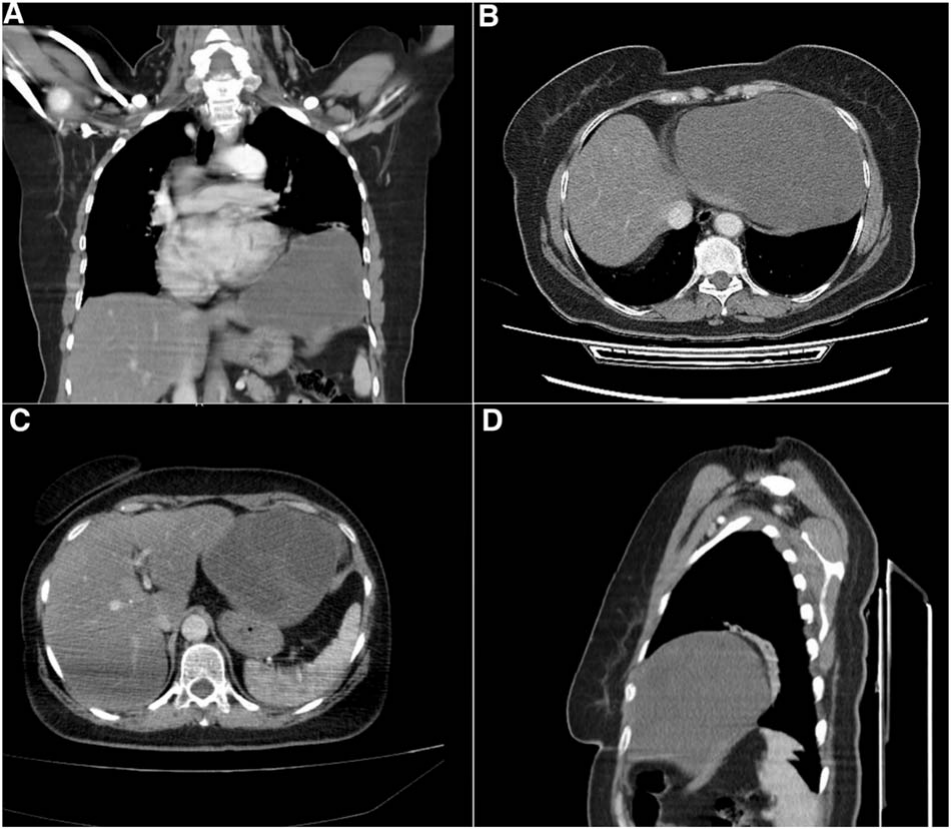
Computed tomography (CT) scan. (A) Coronal section, shows the cystic formation in the left hemi-thorax. (B) and (C) Axial section, shows the borders with the pericardium and the left hepatic lobe. (D) Sagittal section, shows the limited posterior extension of the cyst.

Computed tomography (CT) scan showed a cystic formation containing varying densities. The cyst size was 15 × 13 × 12 cm with an ambiguous location involving the left lower thorax and the left hypochondrium, with a direct bordering of the pericardium and the left hepatic lobe (Fig. 1).

The patient underwent a left posterior-lateral thoracotomy. The cyst was visualized, and dissection was attempted to remove it radically. However, the widespread borders of the cyst, especially with the pericardium, were difficult to reach. The cyst was embedded in the peripheral diaphragm, causing an eventration without penetrating the peritoneum.

**Figure 2:**
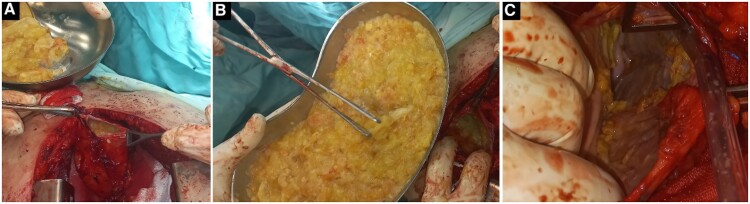
Intraoperative image (A) shows the cyst wall after it was opened and the daughter cysts were being evacuated. (B) A huge number of necrotized daughter cysts. (C) The cyst cavity after it was evacuated.

**Figure 3:**
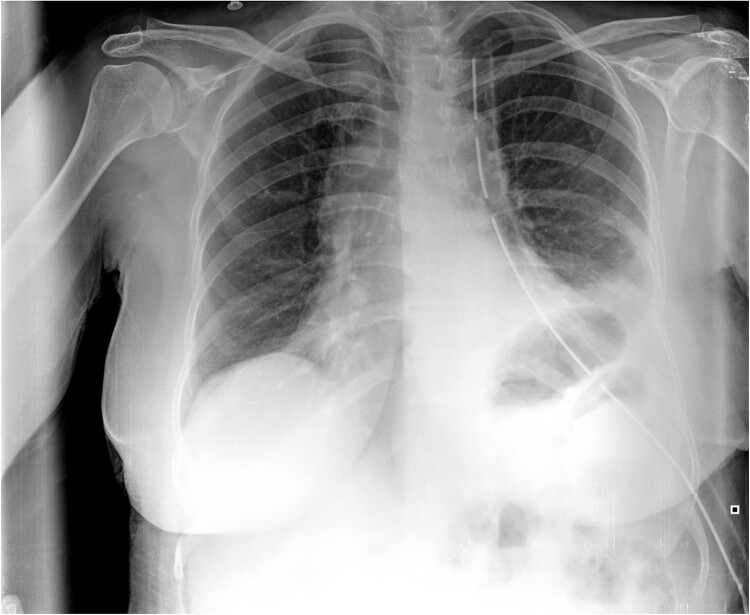
Postoperative CXR. It shows partial lung expansion with apical chest tube.

We performed a cystotomy and evacuation of the cystic cavity, followed by washing it with povidone and hyperosmolar saline. It contained an enormous amount of daughter cysts. The diaphragm defect was small, so it was closed without a mesh to repair the eventration. Then, the diaphragm was sutured to the ribs to give it more stability. Two drainages were put in the cyst and thoracic cavities. Albendazole 10 mg/kg was administered orally for 6 months postoperatively to avoid recurrence. After 12 months of follow-up, no recurrence was observed (Figs 2 and 3).

**Figure 4:**
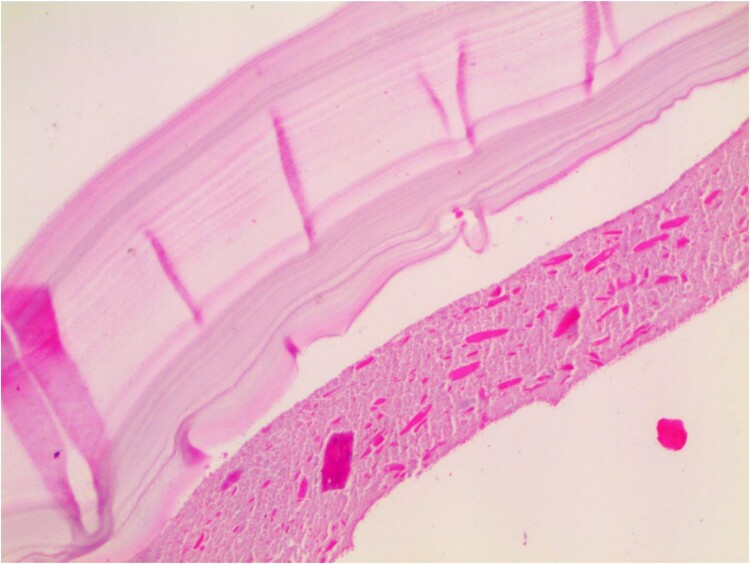
Histopathological examination: the cyst wall comprises an acellular laminated membrane and an inner nucleated germinal layer. Within the cystic cavity, necrotic debris is predominantly present. (Hematoxylin and eosin staining; 100× magnification.)

Histopathological examination revealed that the cyst wall comprises an acellular laminated membrane and an inner nucleated germinal layer. Within the cystic cavity, necrotic debris is predominantly present (Fig. 4).

## Discussion

HC disease, a parasitic disease caused by *Echinococcus granulosus*, is still considered to be endemic in many countries in the Mediterranean region [1]. It affects humans by accidentally ingesting eggs excreted in the feces of primary hosts, such as dogs or sheep [5]. Many organs of the body could be affected; however, intrathoracic extrapulmonary HCs are very rare [4].

There are three main pathways for the development of intrathoracic HCs [6]. In the first pathway, embryos reach the small intestine’s lymphatic system, travel through the thoracic duct and the internal jugular vein to the right side of the heart, and then to the lungs. In the second pathway, embryos after adhering and penetrating to the duodenum and jejunum mucosa, they reach to the mesenteric venules, and move to the portal vein. In the portal system, certain embryos that are smaller than 0.3 mm in diameter may be able to pass through the sinus capillaries of the liver and then travel through the hepatic vein and vena cava to proceed to the right side of the heart and then to the pulmonary vessels. The third pathway is a venal-venous anastomosis in the liver.

HCs may cause a variety of symptoms due to lung compression, such as chest pain, cough, and dyspnea [7]. In many cases, they may be asymptomatic and are diagnosed incidentally on radiological tests conducted for other reasons.

The primary diagnostic tests of HCs are radiological and serological tests. It is worth noting that serological tests, such as the *Echinococcus granulosus* IgG serum anti-bodies test lacks sensitivity and specificity and may yield false negative results, as in our case [8]. Reasons for false negative results may be explained based on many factors, including cyst location other than the liver, hydatid fluid antigenic source variability, and early inactive cyst stages [8].

Due to the rare location of intrathoracic extrapulmonary HCs, the preoperative diagnosis may be challenging, requiring the accurate diagnosis to be confirmed pathologically and the precise location of the cysts to be verified during surgery. A chest CT scan and abdominal echography gave the impression that the cyst extended to the left hypochondrium. During surgery, no cystic formation in the abdomen was observed. The top differential diagnosis was morgani hernia. Also, diaphragmatic, neurenteric, and neurentric duplication cysts were put into consideration.

The complete surgical removal of intrathoracic extrapulmonary HCs is the treatment of choice to avoid recurrence [9]. Nevertheless, due to the location of the cyst and its widespread border with the pericardium at the left cadiophrenic angle, we opted to do a cystotomy and evacuate the cavity contents of the daughter cysts. The cyst was embedded in the diaphragm, causing eventration without penetrating the peritoneum. After repairing the diaphragm, it was sutured to the ribs to provide stability and proper lung inflation.

The main particularities of our case are the location and borders of the cyst involving the left cardiophrenic angle and causing eventration of the diaphragm. The appropriate surgical approach to HCs should be made based on many factors, such as the patient’s status, the location, borders, and size of the cyst.

## Conclusion

Intrathoracic extrapulmonary HCs causing eventration are very rare. To our knowledge, this is the first case report of a HC combining those two occurrences involving the left cardiophrenic angle. Complete surgical resection is the standard treatment for intrathoracic extrapulmonary HCs. The location of the cyst has an important role in determining the surgical approach, as the unusual locations could affect the possibility of radically removing the cyst. HCs, especially in endemic areas, should always be a differential diagnosis for patients presenting with a cyst lesion in the thoracic cavity.

## References

[1] Gessese AT . Review on epidemiology and public health significance of hydatidosis. Vet Med Int 2020;2020:1–8. 10.1155/2020/8859116. PMC7735834 33354312

[2] Abbasi B , Akhavan R, Ghamari A, et al. Heliyon computed tomography and magnetic resonance imaging of hydatid disease: a pictorial review of uncommon imaging presentations. Heliyon 2021;7:e07086. 10.1016/j.heliyon.2021.e07086. 34095581 PMC8166760

[3] Moro P , Schantz PM. Echinococcosis: a review. Int J Infect Dis IJID Off Publ Int Soc Infect Dis 2009;13:125–33. 10.1016/j.ijid.2008.03.037. 18938096

[4] Oǧuzkaya F , Akçah Y, Kahraman C, et al. Unusually located hydatid cysts: intrathoracic but extrapulmonary. Ann Thorac Surg 1997;64:334–7. 10.1016/S0003-4975(97)00521-3. 9262570

[5] Lewall DB . Hydatid disease: biology, pathology, imaging and classification. Clin Radiol 1998;53:863–74. 10.1016/S0009-9260(98)80212-2. 9867269

[6] Isitmangil T , Toker A, Sebit S, et al. A novel terminology and dissemination theory for a subgroup of intrathoracic extrapulmonary hydatid cysts. Med Hypotheses 2003;61:68–71. 10.1016/S0306-9877(03)00108-7. 12781644

[7] Gursoy S , Ucvet A, Tozum H, et al. Primary intrathoracic extrapulmonary hydatid cysts. Tex Heart Inst J 2009;36:230–3. 19568393 PMC2696497

[8] Manzano-Román R , Sánchez-Ovejero C, Hernández-González A, et al. Serological diagnosis and follow-up of human cystic echinococcosis: a new hope for the future? Biomed Res Int 2015;2015:1–9. 10.1155/2015/428205. PMC4609352 26504805

[9] Machboua A , Elhani FZ, Marouf R. Intra thoracic extra pulmonary hydatidosis: prognosis and outcomes of 8 operated patients. J Cardiothorac Surg 2023;18:24–8. 10.1186/s13019-023-02115-6. 36642711 PMC9841658

